# Supplementary material for: Rare Genomic Copy Number Variants Implicate New Candidate Genes for Bicuspid Aortic Valve
Source: medRxiv. 2023 Oct 24:2023.10.23.23297397. Preprint. [Version 1] doi: 10.1101/2023.10.23.23297397 (PMC10635161; doi:10.1101/2023.10.23.23297397)
Supplement: Supplement 1 [file NIHPP2023.10.23.23297397v1-supplement-1.pdf]

medRxiv preprint doi: https://doi.org/10.1101/2023.10.23.23297397; this version posted October 24, 2023. The copyright holder for this preprint (which was not certified by peer review) is the author/funder, who has granted medRxiv a license to display the preprint in perpetuity.
It is made available under a CC-BY 4.0 International license .

Supplemental Data

Cohort	Study	Samples	Accession	Microarray
**WLS**	Wisconsin Longitudinal Study on Aging	8969	Phs001157.v1.pl	Illumina HumanOmniExpress-24 v1.1
**HRS**	Health and Retirement Study	9426	phs000428.v2.pl	Illumina Human Omni2.5-Quad

S1 Table. Summary of Control Cohorts. Cohort, name of control cohort. Study, study from which genotypes were obtained. Samples, number of control samples in each dataset. Accession, Database of Genotypes and Phenotypes accession number. Microarray, Illumina microarray used for genotyping.

	EBAV	BAVGWAS	WLS	HRS
**PennCNV**	6781	73784	58115	163938
**cnvPartition**	2289	33640	31148	51794
**QuantiSNP**	1798	21326	14346	85312
**Merged**	902	7622	21343	14657
**Deletions**	610	2772	8170	6770
**>5 MB**	9	22	9830	6114
**Rare**	84	579	1443	1372
**Rare Deletions**	59	181	285	394

S2 Table. Comprehensive CNV Summary.EBAV, EBAV Cohort including cases and unaffected family members. BAVGWAS, BAVGWAS cohort. WLS, WLS cohort. HRS, HRS cohort. PennCNV, number of CNV calls detected by PennCNV algorithm after quality control. cnvPartition, number of CNV calls detected by cnvPartition algorithm after quality control. QuantiSNP, number of CNVs detected by QuantiSNP algorithm after quality control. Merged, number of CNV regions after merging initial calls. Deletions, number of CNV regions that are deletions. >5 MB, number of CNV regions that are larger than 5 megabases. Rare, number of large (> 250 kilobases and less than 5 megabases) CNV regions that occur in less than 1 in 1000 samples based on case-control cohort pairs (EBAV and WLS; BAVGWAS and HRS). R. Del., number of large, rare deletions. All values reflect the total CNV calls and regions prior to validation in GenomeStudio.

Chr.	Start BP	Stop BP	Type
1	187296703	187609850	DEL
1	146326373	147340734	DUP
1	79238015	79619893	DEL
1	228625778	228880626	DUP
2	114458921	115208197	DUP
2	4638261	5564549	DUP
3	31901848	32165994	DUP
3	19363589	19813225	DEL
4	84658825	85270309	DUP
5	25468811	25719474	DEL
5	78016365	78286867	DUP
6	95836160	96095769	DEL
8	89353386	89800669	DEL

medRxiv preprint doi: https://doi.org/10.1101/2023.10.23.23297397; this version posted October 24, 2023. The copyright holder for this preprint (which was not certified by peer review) is the author/funder, who has granted medRxiv a license to display the preprint in perpetuity.
It is made available under a CC-BY 4.0 International license .

8	2319555	2585105	DUP
8	4201652	4493979	DUP
8	10111571	10721128	DUP
8	11103895	11856864	DUP
8	9368431	9745798	DUP
8	11448529	11732454	DUP
8	11448529	11808756	DUP
10	134505252	135203544	DEL
12	84108147	84443245	DUP
13	70578273	71593281	DUP
15	32908301	34761123	DEL
16	83302526	84016062	DUP
17	1389	582832	DEL
18	57590566	57955945	DUP
21	41268738	41813285	DUP
21	41268738	41823356	DUP
21	41278694	41823356	DUP
21	41278694	41823356	DUP
22	19580050	20227551	DUP
22	46261909	46931077	DEL
22	48871294	51187440	DEL

S3 Table. Large, Rare Copy Number Variants Identified in the EBAV Cohort. Chr., Chromosome on which CNV is located. Start BP, start basepair of CNV. Stop BP, stop basepair of CNV. Type, denotes if a CNV is a duplication (DUP) or deletion (DEL) event. All CNVs were validated in GenomeStudio.

Gene(s)	Chr.	Start BP	Stop BP	Type
HYDIN2, NBPF12, LOC728989, NBPF13P, PRKAB2, PDIA3P, FM05, CHD1L, LINC00624, BCL9, ACP6,* and *GJA5	1	146326373	147340734	DUP
HYDIN2, NBPF12, LOC728989, NBPF13P, PRKAB2, PDIA3P, FM05, CHD1L, LINC00624, BCL9, ACP6,* and *GJA5*	1	146326373	147229299	DUP
MIR4782, SLC35F5, ACTR3, LOC100499194,* and *LOC440900*	2	114426115	115208197	DUP
MIR4782, SLC35F5, ACTR3, LOC100499194,* and *LOC440900*	2	114614021	114732241	DUP
MIR4782, SLC35F5, ACTR3, LOC100499194,* and *LOC440900*	2	114458921	115208197	DUP
MIR4782, SLC35F5, ACTR3, LOC100499194,* and *LOC440900	2	114458921	115208197	DUP
TTN, AX746670, TTN-AS1,* and *MIR548N	2	179364778	179486671	DUP

medRxiv preprint doi: https://doi.org/10.1101/2023.10.23.23297397; this version posted October 24, 2023. The copyright holder for this preprint (which was not certified by peer review) is the author/funder, who has granted medRxiv a license to display the preprint in perpetuity.
It is made available under a CC-BY 4.0 International license .

TTN, AX746670, TTN-AS1,* and *MIR548N*	2	179395466	179517632	DUP
GATA4, C8orf49, NEIL2, FDFT1,* and *CTSB	8	11506208	11786255	DUP
GATA4, C8orf49, NEIL2, FDFT1,* and *CTSB	8	11103895	11856864	DUP
GATA4, C8orf49, NEIL2, FDFT1,* and *CTSB	8	11448529	11808756	DUP
GATA4, C8orf49, NEIL2, FDFT1,* and *CTSB	8	11448529	11732454	DUP
PARD3	10	35107733	35284461	DUP
PARD3	10	35107733	35271898	DUP
KLHL1* and *ATXN8OS	13	70578273	71593281	DUP
KLHL1* and *ATXN8OS*	13	70589082	71548725	DUP
KLHL1* and *ATXN8OS*	13	70730307	70773605	DEL
NECAB2	16	83302526	84016062	DUP
NECAB2*	16	83303915	83999565	DUP
PCP4, DSCAM, MIR4760,* and *DSCAM-AS1	21	41278694	41823356	DUP
PCP4, DSCAM, MIR4760,* and *DSCAM-AS1	21	41268738	41813285	DUP
PCP4, DSCAM, MIR4760,* and *DSCAM-AS1	21	41278694	41823356	DUP
PCP4, DSCAM, MIR4760,* and *DSCAM-AS1	21	41278694	41813285	DUP
PCP4, DSCAM, MIR4760,* and *DSCAM-AS1	21	41268738	41823356	DUP
TBX1, GNB1L, C22orf29, TXNRD2, COMT, MIR4761, ARVCF, TANGO2, MIR185, DGCR8, MIR3618, MIR1306, TRMT2A, RANBP1, ZDHHC8, LOC388849, LOC284865,* and *LINC00896	22	19580050	20227551	DUP
TBX1, GNB1L, C22orf29, TXNRD2, COMT, MIR4761, ARVCF, TANGO2, MIR185, DGCR8, MIR3618, MIR1306, TRMT2A, RANBP1, ZDHHC8, LOC388849, LOC284865, LINC00896, RTN4R,* and *MIR1286	22	18877787	21461607	DUP

S4 Table. Rare CNVs Enriched in EBAV Cohort. Gene(s), genes intersected by CNV. Chr, chromosome on which each CNV is on. Start BP, start basepair of each CNV. Stop BP, stop basepair of each CNV. Type, denotes if a CNV was a duplication (DUP) or deletion (DEL) event.

medRxiv preprint doi: https://doi.org/10.1101/2023.10.23.23297397; this version posted October 24, 2023. The copyright holder for this preprint (which was not certified by peer review) is the author/funder, who has granted medRxiv a license to display the preprint in perpetuity.
It is made available under a CC-BY 4.0 International license .

* Indicates the call was from an unaffected family member.

Gene(s)	Chr	Start BP	Stop BP	Type
LOC100507334	2	110852875	111406073	DUP
LOC100507334	2	110982530	112007875	DUP
MIR128-2	3	35775249	35938795	DUP
MIR128-2	3	35775249	35938795	DUP
MIR128-2	3	35785608	35936616	DUP
TMPRSS11E, UGT2B17, UGT2B15, UGT2B10	4	69599357	69712995	DUP
AHRR, C5orf55, EXOC3, FLJ00157, AK023178, PP7080, BC013821, LOC100996325, and *CEP72*	5	323965	889536	DUP
AHRR, C5orf55, EXOC3, FLJ00157, AK023178, PP7080, and *BC013821*	5	287907	602256	DUP
AHRR, C5orf55, EXOC3, FLJ00157, AK023178, PP7080, and *BC013821*	5	310925	548342	DUP
AHRR, C5orf55, EXOC3, FLJ00157, AK023178, PP7080, BC013821, LOC100996325, and *CEP72*	5	426109	673408	DUP
AHRR, C5orf55, EXOC3, FLJ00157, AK023178, PP7080, BC013821, LOC100996325, and *CEP72*	5	589727	701920	DUP
AHRR, C5orf55, EXOC3, FLJ00157, AK023178, PP7080, BC013821, LOC100996325, and *CEP72*	5	589727	701920	DUP
NIPBL	5	36764235	37046626	DUP
NIPBL	5	36805679	37046626	DUP
NIPBL	5	36898424	37046626	DUP
NIPBL	5	36911625	37052624	DUP
SGK223, CLDN23, and *MFHAS1*	8	8064756	11143272	DUP
SGK223, CLDN23, and *MFHAS1*	8	8064756	11882065	DUP
SGK223, CLDN23, and *MFHAS1*	8	8064756	8655355	DUP
SGK223, CLDN23, and *MFHAS1*	8	8114228	8627839	DUP
SGK223, CLDN23, and *MFHAS1*	8	8202294	8674049	DUP
SGK223, CLDN23, and *MFHAS1*	8	8221088	8650456	DUP
CUL5	11	107755731	107965390	DUP
NANOG and *NANOGNB*	12	7893437	8101326	DUP

medRxiv preprint doi: https://doi.org/10.1101/2023.10.23.23297397; this version posted October 24, 2023. The copyright holder for this preprint (which was not certified by peer review) is the author/funder, who has granted medRxiv a license to display the preprint in perpetuity.
It is made available under a CC-BY 4.0 International license .

NANOG* and *NANOGNB	12	7918339	8109412	DUP
NANOG* and *NANOGNB	12	7942473	8109412	DUP
NANOG* and *NANOGNB	12	7942945	8123777	DUP
NANOG* and *NANOGNB	12	7942945	8105015	DUP
NANOG* and *NANOGNB	12	7945559	8101326	DUP
NANOG* and *NANOGNB	12	7945559	8105015	DUP
NANOG* and *NANOGNB	12	7945559	8105015	DUP
NANOG* and *NANOGNB	12	7945559	8109412	DUP
NANOG* and *NANOGNB	12	7945559	8130958	DUP
UBE2MP1, LOC283914, LOC146481,* and *LOC100130700	16	34355747	34740580	DUP
LOC283914* and *LOC146481	16	34428972	34723621	DUP
LOC283914	16	34433468	34663346	DUP
FAM101B, VPS53,* and *FAM57A	17	1389	641023	DUP
FAM101B, VPS53, FAM57A, GEMIN4, DQ581337,* and *DBIL5P	17	225778	906268	DEL
FAM101B, VPS53, FAM57A,* and *GEMIN4	17	225778	649766	DUP
FAM101B, VPS53, FAM57A,* and *GEMIN4	17	238906	650372	DUP
FAM101B, VPS53, FAM57A, GEMIN4, DQ581337,* and *DBIL5P	17	284614	831667	DUP
RYR1, MAP4K1,* and *EIF3K	19	38683266	39116961	DUP
RYR1, MAP4K1,* and *EIF3K	19	38976659	39116961	DUP
RYR1, MAP4K1,* and *EIF3K	19	38993142	39116961	DUP
RYR1, MAP4K1,* and *EIF3K	19	38993142	39116961	DUP
CYP2A7, CYP2G1P, CYP2B7P1,* and *CYP2B6	19	41349732	41508557	DUP
CYP2A7, CYP2G1P, CYP2B7P1,* and *CYP2B6	19	41350509	41600054	DUP
CYP2A7, CYP2G1P, CYP2B7P1,* and *CYP2B6	19	41354458	41588347	DUP
CYP2A7, CYP2G1P, CYP2B7P1,* and *CYP2B6	19	41386035	41522338	DUP
CYP2A7, CYP2G1P, CYP2B7P1,* and *CYP2B6	19	41386814	41531705	DUP
CYP2A7, CYP2G1P, CYP2B7P1,* and *CYP2B6	19	41386814	41519306	DUP

S5 Table. Rare CNVs Enriched in BAVGWAS Cohort. Gene(s), genes intersected by CNV. Chr, chromosome on which each CNV is on. Start BP, start basepair of each CNV. Stop BP, stop basepair of each CNV. Type, denotes if a CNV was a duplication (DUP) or deletion (DEL) event.

medRxiv preprint doi: https://doi.org/10.1101/2023.10.23.23297397; this version posted October 24, 2023. The copyright holder for this preprint
(which was not certified by peer review) is the author/funder, who has granted medRxiv a license to display the preprint in perpetuity.
It is made available under a CC-BY 4.0 International license .

Principal Gene/Regions	Chr.	Start BP	Stop BP	Type
KIF1A	2	241640262	241678528	DUP
KIF1A	2	241640262	241678528	DUP
KIF1A	2	241652252	241678528	DUP
*KIF1A**	2	241626057	241702124	DUP
*KIF1A**	2	241607616	241702124	DUP
*KIF1A**	2	241644718	241709924	DUP
LTBP1	2	32639775	33331219	DUP
LTBP1	2	32775984	33331219	DUP
*LTBP1**	2	32633925	33331219	DUP
*LTBP1**	2	32633925	33331219	DUP
*LTBP1**	2	32639775	33331219	DUP
RAF1	3	12599717	12803792	DUP
*FLT4**	5	180019198	180056863	DEL
MICA	6	31360255	31453029	DEL
MICA	6	31360255	31485928	DEL
MICA	6	31360255	31487876	DEL
MICA	6	31360255	31457633	DUP
MICA	6	31361397	31453029	DUP
*MICA**	6	31360255	31453029	DEL
*MICA**	6	31360255	31453029	DEL
*MICA**	6	31360255	31453029	DEL
*MICA**	6	31360255	31485928	DEL
*MICA**	6	31360255	31485928	DEL
*MICA**	6	31360255	31485928	DEL
*MICA**	6	31383960	31485928	DEL
*MICA**	6	31355260	31453029	DEL
*GATA4***	8	11506208	11786255	DUP
*GATA4***	8	11506208	11999394	DUP
MUC5B	11	1078312	1300406	DUP
*NANOG**	12	7945559	8123777	DUP
MYH11	16	14975292	16295863	DUP
MYH11	16	15484868	18309593	DUP
MAPK3	16	27977483	30174024	DUP
NCOR1	17	15976558	16012829	DUP
*DSCAM***	21	41278161	41856480	DUP
*DSCAM**	21	41278694	41813285	DUP
**22q11***	22	19698129	19883189	DEL
**22q11***	22	19682627	19755127	DEL
**22q11**	22	19701341	19776365	DEL
**22q11**	22	19701341	19808938	DEL

medRxiv preprint doi: https://doi.org/10.1101/2023.10.23.23297397; this version posted October 24, 2023. The copyright holder for this preprint (which was not certified by peer review) is the author/funder, who has granted medRxiv a license to display the preprint in perpetuity.
It is made available under a CC-BY 4.0 International license .

**22q11**	22	20742450	21461607	DEL

S5 Table. EBAV CNVs intersecting with Genes of Interest. Gene/Region, Principal gene or region of interest intersected by CNV. Chr, chromosome on which each CNV is on. Start BP, start basepair of each CNV. Stop BP, stop basepair of each CNV. Type, denotes if a CNV was a duplication (DUP) or deletion (DEL) event.
* Indicates the call was from an unaffected family member.
** Indicates the call was from an affected family member from a multiplex family.

**Principal Gene/Regions**	**Chr.**	**Start BP**	**Stop BP**	**Type**
GJA5	1	145723645	148343177	DUP
GJA5	1	145723739	148343177	DUP
GJA5	1	145801230	147824365	DUP
GJA5	1	147166377	147308112	DUP
TMEM87B/FBLN7	2	110982530	113103748	DUP
TMEM87B/FBLN8	2	111399346	113103748	DEL
TMEM87B/FBLN9	2	111404636	113215796	DUP
KIF1A	2	241623458	241697884	DUP
KIF1A	2	241623458	241697884	DUP
KIF1A	2	241623458	241698298	DUP
KIF1A	2	241623458	241724479	DUP
KIF1A	2	241626057	241689833	DUP
KIF1A	2	241626057	241689833	DUP
KIF1A	2	241626057	241689833	DUP
KIF1A	2	241626057	241689833	DUP
KIF1A	2	241626057	241689833	DUP
KIF1A	2	241626057	241702124	DUP
KIF1A	2	241626057	241702124	DUP
KIF1A	2	241640262	241689833	DUP
KIF1A	2	241640262	241697773	DUP
LTBP1	2	32619581	33299434	DUP
LTBP1	2	32619581	33331219	DUP
LTBP1	2	32633925	33302342	DUP
LTBP1	2	32633925	33302342	DUP
LTBP1	2	32633925	33331219	DUP
LTBP1	2	32633925	33331219	DUP
LTBP1	2	32633925	33331219	DUP
LTBP1	2	32633925	33369552	DUP
LTBP1	2	32689829	33331219	DUP
RAF1	3	12645681	12739194	DUP
TGFBR2	3	29993977	31273870	DEL
**SOX7/GATA4**	8	8064756	11882065	DUP
**SOX7/GATA4**	8	8064756	11882065	DUP
**SOX7/GATA4**	8	8064756	11882065	DUP

medRxiv preprint doi: https://doi.org/10.1101/2023.10.23.23297397; this version posted October 24, 2023. The copyright holder for this preprint (which was not certified by peer review) is the author/funder, who has granted medRxiv a license to display the preprint in perpetuity.
It is made available under a CC-BY 4.0 International license .

SOX7/GATA4	8	8064756	12009597	DUP
SOX7/GATA4	8	10109379	11987960	DUP
SOX7	8	10587741	10683929	DEL
GATA4	8	10914233	11853596	DUP
GATA4	8	11349186	11821835	DUP
GATA4	8	11385469	11882065	DUP
TGFBR1	9	101861767	102092282	DUP
MYH11	16	14761719	16281154	DUP
MYH11	16	14761719	16315360	DUP
MYH11	16	14975292	16299148	DEL
MYH11	16	14975292	16308351	DUP
MYH11	16	14975292	16308351	DUP
MYH11	16	14975292	16308351	DUP
MYH11	16	14975292	16308351	DUP
MYH11	16	14975292	16308351	DUP
MYH11	16	14975292	16308351	DUP
MYH11	16	14975292	16315360	DUP
MYH11	16	15092120	16291933	DUP
MYH11	16	15125441	16292128	DUP
MYH11	16	15240816	18584353	DUP
MAPK3	16	29647342	30199713	DUP
MAPK4	16	29647342	30199713	DUP
MAPK5	16	29647342	30199713	DUP
DSCAM	21	41254102	41516071	DUP
DSCAM	21	41254456	41536215	DUP
**22q11**	22	16874656	20241436	DEL
**22q11**	22	17818807	19002159	DUP
**22q11**	22	18644702	21726191	DUP
**22q11**	22	18877787	21461607	DUP
**22q11**	22	18877787	21461607	DUP
**22q11**	22	18877787	21028007	DEL
**22q11**	22	18877787	21804903	DEL
**22q11**	22	19062020	20264937	DUP
**22q11**	22	19667336	20329526	DEL
**22q11**	22	19682627	20233865	DEL
**22q11**	22	19682627	20262166	DEL
**22q11**	22	19693418	20264937	DEL
**22q11**	22	19701341	20300738	DEL
**22q11**	22	19724224	20300738	DEL
**22q11**	22	19951816	24298181	DUP
**22q11**	22	20719325	21726191	DEL

medRxiv preprint doi: https://doi.org/10.1101/2023.10.23.23297397; this version posted October 24, 2023. The copyright holder for this preprint (which was not certified by peer review) is the author/funder, who has granted medRxiv a license to display the preprint in perpetuity.
It is made available under a CC-BY 4.0 International license .

**22q11**	22	21246902	22702508	DEL
**22q11**	22	21424414	22015771	DUP
CESLR1	22	45236935	48193505	DEL
CESLR1	22	46751367	47159028	DUP
CESLR1	22	46924254	46931077	DEL

S7 Table. BAVGWAS CNVs intersecting with Genes of Interest. Gene/Region, Principal gene or region of interest intersected by CNV. Chr, chromosome on which each CNV is on. Start BP, start basepair of each CNV. Stop BP, stop basepair of each CNV. Type, denotes if a CNV was a duplication (DUP) or deletion (DEL) event.

Chr.	Start BP	Stop BP	Type	Description
**2**	138066736	143331537	DUP	Mosaic LOH
**2**	183476298	189945752	DUP	Mosaic LOH
**3**	143040791	168814375	DUP	Mosaic LOH
**3**	66206	7768285	DEL	Constitutional
**6**	148301116	156618923	DEL	Constitutional
**7**	101355402	106892492	DEL	Mosaic
**8**	6970806	12525566	DUP	Constitutional
**8**	170692	11987960	DUP	Constitutional
**14**	101350298	107283150	DUP	Mosaic LOH
**14**	71135027	107283150	DUP	Mosaic LOH
**15**	80465431	88497147	DUP	Mosaic LOH
**15**	93593528	102150818	DUP	Mosaic LOH
**15**	22761722	28540261	DEL	Constitutional
**17**	15175570	22234751	DUP	Mosaic
**18**	67445173	78010620	DEL	Constitutional
**20**	31265482	50716159	DEL	Mosaic
**20**	61098	25829977	DEL	Mosaic
**20**	31240778	48292606	DEL	Mosaic
**20**	50320079	62960292	DUP	Constitutional
**21**	14359894	48099610	DUP	Trisomy 21
**21**	14359894	48099610	DUP	Trisomy 21

S8 Table. Large Genomic Events in BAVGWAS Chr., Chromosome CNV on which CNV is located. Start BP, start base pair of CNV. Stop BP, stop base pair of CNV. Type, denotes if a CNV is a duplication (DUP) or deletion (DEL) event. Description, denotes if the CNV was a mosaic loss of heterozygosity (Mosaic LOH), loss of heterozygosity (LOH), mosaic (Mosaic), constitutional (constitutional), or trisomy 21 (Trisomy 21) event.

PROBAND	GENE	SEGREGATES?	WITH CNV	NO CNV	SEX
**BAV064**	*GATA4*	Yes	Father*, Paternal Grandfather*	Paternal Grandmother	Female
**BAV475**	*DSCAM*	Yes	Sister*	Father	Female
**BAV787**	*CELSR1*	Yes	None	Daughter	Female

medRxiv preprint doi: https://doi.org/10.1101/2023.10.23.23297397; this version posted October 24, 2023. The copyright holder for this preprint (which was not certified by peer review) is the author/funder, who has granted medRxiv a license to display the preprint in perpetuity.
It is made available under a CC-BY 4.0 International license .

**BAV330**	*KIF1A*	Yes	None	Father	Female
**BAV478**	*KIF1A*	Yes	None	Father	Male
**BAV829**	*LTBP1*	No	Son, Father	Mother, Brother	Female

S9 Table. Pedigree Information for CNVs that Segregated with Disease. Proband, identification number of proband with CNV intersecting with gene of interest. Gene, gene of interest intersected by CNV. Segregates?, indicates if the CNV segregated with disease. Family With CNV, family members of proband that were found to have a CNV intersecting with the respective gene. Family Without CNV, family members of proband who were not found to have a CNV intersecting with the respective gene. Family members are listed if their genotype was available for the study. Sex, sex of the proband.
*Indicates family members who also have BAV.
